# Human cytomegalovirus seropositivity is associated with reduced patient survival during sepsis

**DOI:** 10.1186/s13054-023-04713-1

**Published:** 2023-10-31

**Authors:** M. Unterberg, S. F. Ehrentraut, T. Bracht, A. Wolf, H. Haberl, A. von Busch, K. Rump, D. Ziehe, M. Bazzi, P. Thon, B. Sitek, K. Marcus, M. Bayer, K. Schork, M. Eisenacher, B. Ellger, D. Oswald, F. Wappler, J. Defosse, D. Henzler, T. Köhler, A. Zarbock, C. P. Putensen, J. C. Schewe, U. H. Frey, M. Anft, N. Babel, E. Steinmann, Y. Brüggemann, M. Trilling, A. Schlüter, H. Nowak, M. Adamzik, T. Rahmel, B. Koos, SepsisDataNet.NRW research group

**Affiliations:** 1 Klinik für Anästhesiologie, Intensivmedizin und Schmerztherapie, Universitätsklinikum Knappschaftskrankenhaus Bochum, Bochum, Germany; 2 Klinik für Anästhesiologie und Operative Intensivmedizin, Universitätsklinikum Bonn, Bonn, Germany; 3 Medizinisches Proteom-Center, Ruhr-University Bochum, 44801 Bochum, Germany; 4 Medical Proteome Analysis, Center for Proteindiagnostics (PRODI), Ruhr University Bochum, 44801 Bochum, Germany; 5 Klinik für Anästhesiologie, Intensivmedizin und Schmerztherapie, Klinikum Westfalen, Dortmund, Germany; 6 Department of Anaesthesiology and Operative Intensive Care Medicine, University of Witten/Herdecke, Cologne Merheim Medical School, Cologne, Germany; 7 Department of Anesthesiology, Surgical Intensive Care, Emergency and Pain Medicine, Ruhr-University Bochum, Klinikum Herford, Herford, Germany; 8 Department of Anesthesiology and Intensive Care Medicine, AMEOS-Klinikum Halberstadt, Halberstadt, Germany; 9 Klinik für Anästhesiologie, Operative Intensivmedizin und Schmerztherapie, Universitätsklinikum Münster, Münster, Germany; 10 Marien Hospital Herne, Universitätsklinikum der Ruhr-Universität Bochum, Bochum, Germany; 11 Center for Translational Medicine, Medical Clinic I, Marien Hospital Herne, University Hospital of the Ruhr-University Bochum, Herne, Germany; 12 Department of Molecular and Medical Virology, Ruhr University Bochum, 44801 Bochum, Germany; 13 Institute for Virology, University Hospital Essen, University of Duisburg-Essen, Essen, Germany; 14 Knappschaft Kliniken GmbH, Recklinghausen, Germany; 15 Center for Artficial Intelligence, Medical Informatics and Data Science, University Hospital Knappschaftskrankenhaus Bochum, Bochum, Germany

**Keywords:** Sepsis, HCMV latency, Inflammatory biomarker, Mortality prediction

## Abstract

**Background:**

Sepsis is one of the leading causes of death. Treatment attempts targeting the immune response regularly fail in clinical trials. As HCMV latency can modulate the immune response and changes the immune cell composition, we hypothesized that HCMV serostatus affects mortality in sepsis patients.

**Methods:**

We determined the HCMV serostatus (i.e., latency) of 410 prospectively enrolled patients of the multicenter SepsisDataNet.NRW study. Patients were recruited according to the SEPSIS-3 criteria and clinical data were recorded in an observational approach. We quantified 13 cytokines at Days 1, 4, and 8 after enrollment. Proteomics data were analyzed from the plasma samples of 171 patients.

**Results:**

The 30-day mortality was higher in HCMV-seropositive patients than in seronegative sepsis patients (38% vs. 25%, respectively; *p* = 0.008; HR, 1.656; 95% CI 1.135–2.417). This effect was observed independent of age (*p* = 0.010; HR, 1.673; 95% CI 1.131–2.477). The predictive value on the outcome of the increased concentrations of IL-6 was present only in the seropositive cohort (30-day mortality, 63% vs. 24%; HR 3.250; 95% CI 2.075–5.090; *p* < 0.001) with no significant differences in serum concentrations of IL-6 between the two groups. Procalcitonin and IL-10 exhibited the same behavior and were predictive of the outcome only in HCMV-seropositive patients.

**Conclusion:**

We suggest that the predictive value of inflammation-associated biomarkers should be re-evaluated with regard to the HCMV serostatus. Targeting HCMV latency might open a new approach to selecting suitable patients for individualized treatment in sepsis.

**Supplementary Information:**

The online version contains supplementary material available at 10.1186/s13054-023-04713-1.

## Introduction

Sepsis is defined as an acute, life threatening organ dysfunction caused by a dysregulated immune response to a microbial assault [1]. It is one of the leading causes of mortality in industrialized nations, affecting millions of individuals per year [2]. The etiologies of the dysregulated immune response might be diverse, e.g., bacterial assault as well as fungal or viral infection [3, 4]. In addition many confounding factors can influence the immune response such as age, trauma or chronic diseases [5] making the immune response a non-linear, complex system [6]. Nonetheless, it is well recognized that pro- and anti-inflammatory cytokines, such as IL-6 or IL-10, are good predictors of clinical outcome, suggesting the influence of the immune response on the survival of the patient [7–9]. So far, clinical trials that attempted to modulate the dysregulated immune response have largely failed [10, 11]. Recently, the pre-emptive treatment of the human cytomegalovirus (HCMV) has been discussed in the context of sepsis [12, 13].

HCMV is a member of the herpes virus family and is endemic in humans with an increasing rate of seropositivity (IgG +) according to age [14] and hygienic standards [14]. In Europe, the seropositivity rate of HCMV is around 60–70% which is reached in the seventh decade of life [15], rendering HCMV infections relevant in the major parts of the population. While infections in adult immunocompetent individuals frequently progress without overt disease or only mild symptoms, one of the major characteristics of HCMV is its ability to establish latency in the host. Latency is defined by the presence of the replication competent viral DNA in bone marrow hematopoietic progenitor cells [16] and tissue endothelial cells [17] as potential reservoir below the level of immune detection, while most viral translational products/proteins are absent [18]. This state does not cause an active disease. Hence, IgG seropositivity for HCMV is the Gold Standard bio-marker for a latent virus infection as it indicates a prior infection with HCMV and therefore latency of the virus. This latent infection persists for the lifetime of the host [19] and can be followed by a potentially dangerous HCMV reactivation during conditions of impaired immunity [20, 21] which is well known and investigated in numerous trials [20–23]. While the dogma in intensive care medicine is that HCMV reactivation during critical illness is potentially life threatening, little attention has been paid to the latency (i.e., the seropositivity), despite the fact that latency significantly alters the immune response [24] and the composition of the immune system [25].

Therefore, the impact of HCMV latency on the immune system has been suggested and discussed [18] but not sufficiently investigated in critically ill patients. Thus, further research is needed to evaluate the effects of the latent HCMV infection on the hosts’ immunity.

To achieve a state of latency, HCMV counteracts its host immune response in multiple ways. Disturbing the cellular presentation of antigens to T- and NK-cells [26] as well as the modulation of the cellular signaling toward apoptosis [27] preserves the virus’ latent survival but also alters the steady state of the host. An extended cellular surface maintenance of TLR-4 and TLR-5 on CMV-infected cells as well as an amplified intracellular proinflammatory cascade with the phosphorylation of IκB-α and NF-κB and significantly promoted TNF-α, IL-6, and IL-8 expression was described in a macrophage model [28]. These findings suggest a HCMV-induced impairment of the immune system shifting the balance toward a more pronounced inflammation. This might significantly affect the patient when suffering from an infection or even sepsis later in life.

In light of this, we asked (1) if we can find an altered inflammatory response during sepsis associated with the HCMV serostatus and (2) if the HCMV serostatus impacts the 30-day survival of sepsis patients.

## Material and methods

### Study design and cohort

The SepsisDataNet.NRW study [29] (German Clinical Trial Registry No. DRKS00018871; http://www.sepsisdatanet.nrw) prospectively enrolled patients fulfilling the SEPSIS-3 criteria in a multicentric approach from the ICUs of seven different hospitals (university hospitals or tertiary care hospitals) in the German state of North Rhine-Westphalia. This study was approved by the Ethics Committee of the Medical Faculty of Ruhr-University Bochum (Registration No. 18-6606—BR) or the responsible ethics committee of each respective study center. The patients were recruited after obtaining written informed consent from the 1st of March 2018 to the 31th of May 2022. This study included adult patients with a sepsis diagnosis within the previous 36 h according to the current SEPSIS-3 definition (suspected/proven infection and an increase in the Sequential Organ Failure Assessment (SOFA) score by two points or more). The cohort comprised of mixed surgical and medical patients admitted to the ICU. The exclusion criteria were as follows: (1) age of < 18 years old at the time of ICU admission, (2) withdrawal or withhold of consent, and (3) withdrawal of treatment. Patients with unknown 30-day survival status were excluded from further analysis. It is noteworthy that immunosuppression was not an exclusion criterion as we did not focus on re-activation of HCMV.

### Clinical data and patients’ characteristics

Electronic medical data including vitals, laboratory values, point of care diagnostics, demographics, and the length of ICU stay were captured in a comprehensive database (CentraXX software, Kairos GmbH, Bochum, Germany) following pseudonymization according to the obligations of the ethics committee. Missing data was augmented by the individual investigation of patient records at each corresponding clinic by an experienced physician and, where appropriate, completed by including the data from ± 12 h of the onset of sepsis. SAPS-2 DRG as well as SOFA score was manually calculated by an experienced physician at each recruitment site. All patients were treated according to the current sepsis guidelines.

### HCMV serostatus (IgG) and reactivation (IgM and DNA)

#### HCMV IgG and IgM

The patient sera were evaluated for the presence of HCMV IgG at Day 1 and IgM at Days 1 and 8 after enrollment using the SERION ELISA Classic Cytomegalovirus IgG/IgM Kit (Institut Virion\Serion GmbH, Würzburg, Germany). One hundred µL of diluted samples (1:40) and respective controls were applied into micro test wells and incubated for 60 min at 37°C. Afterwards, the samples were washed three times and incubated with 100 µL substrate solution for at least 20 min at 37°C. The reactions were stopped using 100 µL of stopping solution, and the optical densities (OD) were determined using a microplate reader (Sunrise Microplate Reader\Tecan, Maennedorf, Switzerland). The OD values were normalized to a standard and the units were calculated. The IgM samples were classified as positive when 15 or more units were detected, while the IgG assays were classified as positive when 35 or more units were detected.

#### HCMV DNA

Whole blood DNA was used to evaluate the concentration of HCMV DNA at Days 1, 4, and 8. Table 1 shows the primer/probe combination. DNA was subjected to a qPCR analysis using TaqMan Universal Mastermix (Applied Biosystems) in accordance with the manufacturer’s recommendations. The threshold cycles (Ct) were determined and the samples were identified as positive if Ct was < 40. The samples between Ct 35 and 40 were tested a second time to filter out false positives.

**Table 1:** Primer and probe combination for the evaluation of HCMV re-activation

Name	Sequence	Manufacturer
Forward primer	5 ‘-ATAGGAGGCGCCACGTATTC-3 ‘	Biomers
Reverse primer	5 ‘-TACCCCCTATCGCGTGTGTTC-3 ‘	Biomers
Probe	5’-FAM-CGTTTCGTCGTAGCTACGCTTACAT-TAMRA-3’	Biomers

### Cytokine concentrations

As part of the SepsisDataNet.NRW study, the biomaterials (serum) were collected at Days 1, 4, and 8 after recruitment. The serum samples collected at Day 1 were used to quantify the concentration of 13 cytokines at the time of recruitment. The LegendPlex Human Inflammation Panel 1 (Biolegend, San Diego) was used in accordance with the manufacturer’s instructions. Briefly, the serum samples were incubated with LegendPlex beads for antigen capture, washed, and incubated with detection antibodies. After additional washing, the beads were measured using a flow cytometer (Canto II, BD Biosciences, CA), and cytokine concentration was quantified using a standard curve. If the recorded concentration of a cytokine was below the lower limit of detection (LOD), the value was treated as 0 ng/mL. If a value was recorded as higher than the upper LOD, it was treated as the upper LOD.

### Antibodies for flow cytometry

All antibodies are from BioLegend (San Diego) unless otherwise noted: CD16-APC-fire750, clone: 3G8; CD56-AF647, clone: NCAM; CD45RA-BV650, clone: HI100; CCR7-Pe/Dazzle 594, clone: G043H7; CD8-PeCy7, clone: SK1; CD45-A488, clone: SD1; CD4-AF700, clone: OKT4; CD19-BV605, clone: HIB19; CD3-BV785, clone: OKT3; HLADR-PE, clone: L243 (BD Biosciences, CA); CD14-PerCP-Cy5.5, clone: MφP9 (BD Biosciences, CA); Zombie Aqua™ Fixable Viability Kit.

### Immunophenotyping

25 µl EDTA-treated whole blood was stained with 25 µl master mix, containing the optimal concentrations of each antibody, for 10 min at room temperature in the dark. Erythrocytes were lyzed using RBC Lysis Buffer (BioLegend, San Diego) for 10 min at room temperature in the dark and samples were immediately acquired on a CytoFlex flow cytometer (Beckman Coulter, Brea). Quality control was performed daily using the recommended CytoFlex Daily QC Fluorospheres (Beckman Coulter, Brea). No modification to the compensation matrix was required throughout the study.

### Plasma proteomics

The plasma samples from 171 patients who were enrolled in the participating centers in Bochum and Bonn were digested according to the SP3 protocol with slight modifications. Briefly, 100 µg protein was purified using paramagnetic beads (Cytiva Sera-Mag Carboxyl-Magnet-Beads, GE Healthcare, Chicago, IL) and digested overnight using trypsin (SERVA Electrophoresis, Heidelberg, Germany). Subsequently, 300 ng tryptic peptides per sample were analyzed using an Ultimate 3000 RSLCnano HPLC coupled online to either an Orbitrap QExactive, Orbitrap QExactive HF, or Orbitrap Fusion Lumos mass spectrometer (all Thermo Scientific, Bremen, Germany). In total, 306 samples were analyzed and distributed over five batches and separated by either a 96-min (Batch 1) or 38-min (Batches 2–5) LC gradient. The mass spectrometers were operated in data-independent acquisition mode. Spectral libraries were generated with FragPipe (v.17.1) and protein quantification was conducted using DIA-NN (v.1.8) [30]. The Uniprot/SwissProt database restricted to homo-sapiens (release 01_2022; 20,386 entries) was used for protein identification. The resulting protein intensities were first normalized using the LOESS method [31]. The subsequent cross-batch normalization was based on linear regression models. A detailed description of the applied methods can be found in the Additional file.

### Statistics

Statistical analyses were performed using the IBM® SPSS Statistics software version 28. The statistical differences of the categorical variables were determined using Fisher’s Exact Test, while the continuous variables were tested using Student’s t-test and the Mann–Whitney U test. Normally distributed variables were compared using Student’s t-test, while non-normally distributed variables were compared using the Mann–Whitney U test. The impact of a continuous variable on the 30-day survival was analyzed by Receiver Operator Characteristics, Log Rank Tests (visualized by Kaplan–Meier curves), and multivariate Cox regression analysis. First, the best predictive cutoff for each variable was determined by ROC and the calculation of Youden’s index. Accordingly, we defined a cutoff of 440 pg/ml for IL-6, 13.5 pg/mL for IL-10, and 3.43ng/ml for PCT. If not otherwise stated, p-values below 5% were considered significant. Proteomics data were processed using R (v.4.2.1; r-project.org). Statistical differences between the groups were assessed based on normalized protein intensities using Student’s t-test. The resulting p-values were FDR-corrected according to Benjamini-Hochberg. Relative changes were calculated as ratios of means based on delogarithmized intensities.

## Results

### Cohort description

In a multicenter approach, 417 sepsis patients were prospectively recruited between March 2018 and May 2022. Of these, the HCMV serostatus could not be determined for three patients. Furthermore, four patients were lost to follow-up, resulting in a total of 410 patients (250 male, median age 67 [IQR: 57–77] years) included in this analysis.

Overall, 63% of the patients analyzed (259 seropositive vs. 151 seronegative) tested positive for HCMV-IgG (HCMV seropositive) at the time of study inclusion. In total we could identify 21 patients with HCMV activation by PCR (7.5%). Of these 17 patients (81%) were HCMV seropositive indicating a re-activation of the virus with 4 de-novo infections in the sero-negative group (*p* = 0.056). HCMV reactivation determined by IgM was even lower; four patients (1%) were positive for HCMV-specific IgM. The median SOFA score at the time of enrollment was 8 [IQR: 6–12], and the overall 30-day mortality was calculated to be 33% (136 out of 410 patients). With regard to the patient demographics and medical characteristics (age, sex, focus of infection), the seropositive patients were five years older (*p* = 0.004). In both subgroups, the lower respiratory tract and intraabdominal areas were the most frequent focus of infection (Table 2). HCMV seropositive patients had a higher occurrence of transplantation history then seronegative patients (19% vs. 10%, *p* = 0.029).

**Table 2:** Basic characteristics of the cohort divided into the HCMV-seronegative and seropositive groups

	Entire cohort	HCMV-seronegative	HCMV-seropositive	*p*-value	*n*
*n*	410	151	259		410
Male gender *n* (%)	**250 (61%)**	**104 (72%)**	**146 (60%)**	**0.022**	**388**
Age years median [IQR]	**67 [57–77]**	**63 [54–74]**	**68 [59–78]**	**0.004**	**398**
SOFA score median [IQR]	**9 [6–12] **	**8 [5–11]**	**9 [6–12] **	**0.008**	**390**
SAPS-2 DRG median [IQR]	**38 [29–48]**	**34 [26–47]**	**40 [30–48]**	**0.019**	**319**
PCT ng/mL median [IQR]	3.0 [0.6–12.3]	3.0 [0.5–13.0]	3.0 [0.7–12.2]	0.700	317
CRP mg/dL median [IQR]	15 [9–26]	14 [9–26]	16 [9–27]	0.371	299
Lactat mM median [IQR]	1.7 [1.2–3.2]	1.6 [1.1–2.9]	1.8 [1.2–3.3]	0.077	330
Comorbidities *n* (%)	340	124	176		340
Alcohol	27 (8)	10 (8)	17 (8)	1.000	
Chronic kidney disease	85 (25)	28 (23)	57 (26)	0.516	
Hypertension	**214 (63)**	**74 (60)**	**216 (65)**	**0.044**	
Diabetes	87 (26)	25(20)	62 (29)	0.094	
Obesity	83 (24)	28 (23)	55 (25)	0.601	
Cardiovascular	100 (29)	30 (24)	70 (32)	0.138	
Malignancies	105 (31)	42 (34)	63 (29)	0.394	
Nicotine	90 (26)	34 (27)	56 (26)	0.799	
Dialysis	37 (11)	10 (8)	27 (13)	0.278	
Transplantation	**53 (16)**	**12 (10)**	**41 (19)**	**0.029**	
COPD	38 (11)	15 (12)	23 (11)	0.722	
Other (lungs)	24 (7)	11 (9)	13 (6)	0.380	
Focus of infection *n* (%)	362	135	227		362
Central nervous system	9 (3)	6 (3)	3 (1)	0.065	
Lower respiratory tract	161 (45)	64 (47)	97 (43)	0.387	
Genitourinary	25 (7)	11 (8)	14 (6)	0.472	
Intra-abdominal	106 (29)	35 (26)	71 (31)	0.279	
other	61 (17)	19 (14)	42 (19)	0.276	
Leukocytes 10^3^/median [IQR]	14 [9–19]	15 [10–19]	13 [8–20]	0.432	219
Surgical patient *n* (%)	293 (78)	105 (76)	188 (79)	0.444	377
ICU length of stay median days [IQR]	7 [3–15]	7 [3–15]	7 [3–15]	0.557	345
Hospital length of stay median days [IQR]	**18 **[10–34]	**24 **[11–41]	**16 **[9–29]	**0.008**	**317**
30-day mortality *n* (%)	**136 (33)**	**37 (25%)**	**99 (38%)**	**0.005**	**410**

Bold entries are the one that are statistically significant different

Furthermore, in terms of the severity of sepsis, determined by the SOFA score, we found a slightly higher SOFA score (9 vs. 8, *p* = 0.008) in the HCMV-seropositive patients. Table 2 shows the demographic and medical data (Table 2).

### Impact of HCMV serostatus on sepsis mortality

**Fig. 1:**
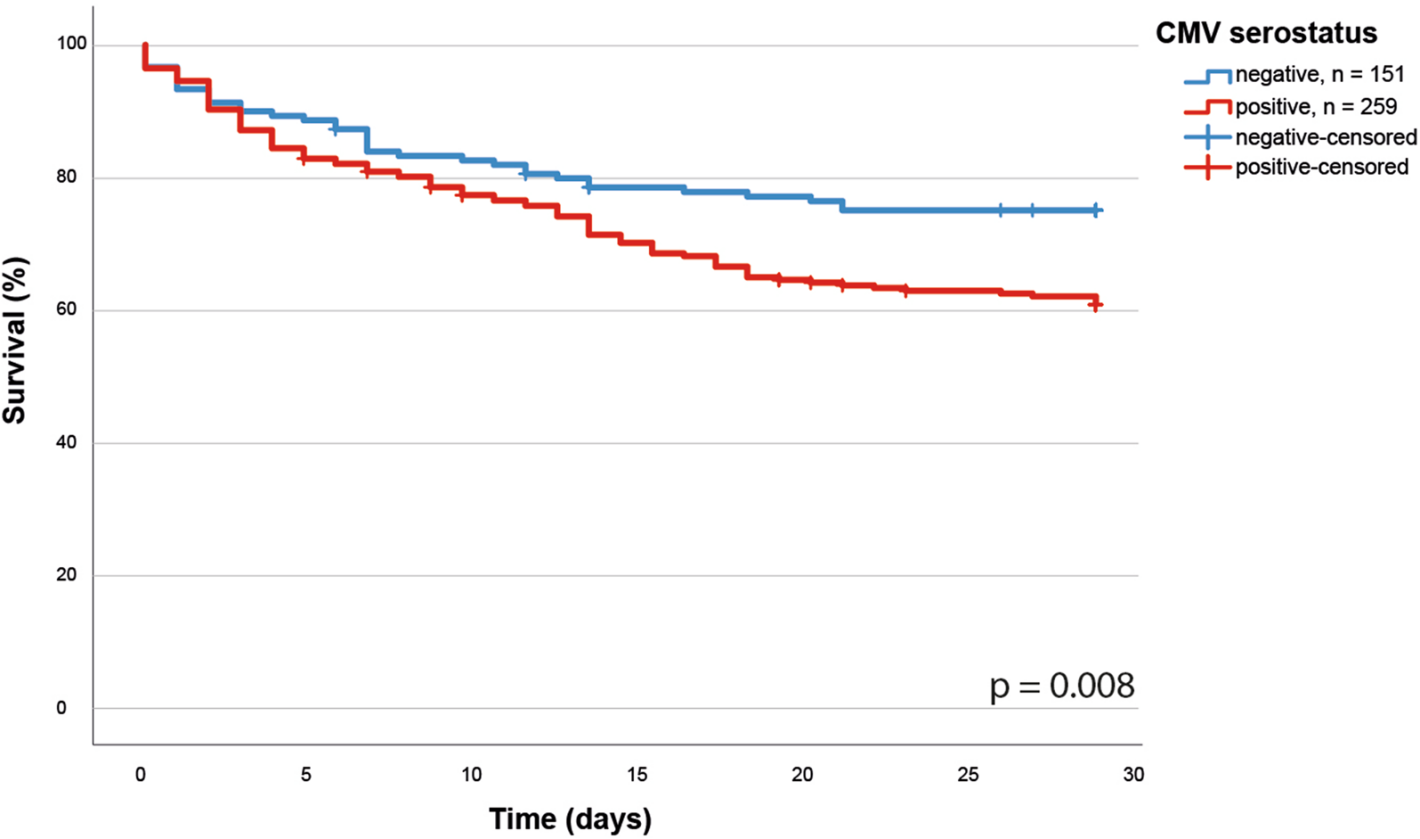
Kaplan–Meier analysis of the impact of HCMV serostatus on the 30-day survival in sepsis

In our cohort we found seropositivity of HCMV to be associated with an increased 30-day mortality, reducing survival by almost 15% (Fig. 1, Kaplan–Meier analysis, *p* = 0.008). The seropositive patients exhibited a significantly reduced 30-day survival compared with seronegative patients (62% vs 76%; *p* = 0.008; HR 1.656; 95% CI 1.135–2.417). Moreover, in a multivariate Cox regression analysis, we found this effect of HCMV seropositivity on 30-day mortality to be independent of age and transplantation co-morbidity (*p* = 0.019; HR 1.638; 95% CI 1.086–2.470) two well know confounders for HCMV seropositivity and the immune response.

### HCMV serostatus does not alter the plasma proteome

The concentrations of pro- and anti-inflammatory cytokines as well as procalcitonin (PCT) at Day 1 were similar for both groups (e.g., IL-10 median seronegative = 6.9 pg/mL [IQR: 14.4] vs. seropositive = 9.3 pg/mL [IQR: 22.7]) (*p* = 0.103) (Table 3). When investigating the patients’ plasma proteome composition at Days 1 and 4, we could not identify any significant changes in the proteome at Day 1, with only minor differences observed at Day 4 (Additional file 3: Tables S1 and Additional file 4: Table S2, Additional file 1: Figure S1). Additional file 5: Table S3 shows the progression of cytokine concentrations in the seronegative and seropositive patients over the three evaluated timepoints.

**Table 3:** Cytokine concentrations (pg/mL) in the sera from seropositive and seronegative patients at study inclusion

	Entire cohort	HCMV-seronegative	HCMV-seropositive	*p*-value
n	334	117	217	
IL-1B median [IQR]	5.1 [2.7–8.9]	5.1 [1.1–9.0]	5.1 [2.7–8.9]	0.649
IFN-α median [IQR]	1.6 [1.2–2.7]	1.6 [1.1–2.4]	1.6 [1.2–2.9]	0.679
IFN-γ median [IQR]	4.0 [0.0–10.2]	4.9 [0.3–10.2]	4.0 [0.0–10.2]	0.647
TNF-α median [IQR]	5.9 [0.0–10.9]	5.3 [0.0–9.3]	6.3 [0.0–11.3]	0.066
MCP-1 median [IQR]	272.9 [147.5–528.7]	248.5 [143.9–466.7]	306.0 [150.4–570.5]	0.242
IL-6 median [IQR]	215.1 [67.4–593.2]	170.1 [71.1–481.2]	259.9 [64.4–654.7]	0.330
IL-8 median [IQR]	**77.9 [31.8–186.2]**	**61.0 [26.9–121.8]**	**84.2 [37.0–199.6]**	**0.004**
IL-10 median [IQR]	7.6 [2.3–23.9]	6.7 [2.2–17.7]	8.2 [2.3–25.6]	0.183
IL-12 median [IQR]	2.8 [0.0–4.4]	2.9 [0.0–4.4]	2.8 [1.5–4.4]	0.693
IL17 median [IQR]	0.5 [0.3–0.9]	0.5 [0.3–0.8]	0.5 [0.3–0.9]	0.177
IL-18 median [IQR]	287.0 [138.6–699.3]	225.8 [115.7–548.2]	345.1 [153.3–771.4]	0.009
IL-23 median [IQR]	6.5 [0.0–27.5]	10.7 [0.0–30.3]	6.1 [0.0–25.4]	0.145
IL-33 median [IQR]	23.0 [9.7–46.5]	25.6 [12.0–47.5]	22.5 [9.7–46.5]	0.743

*p*-values of < 0.004 (Bonferroni correction) are considered significant

Bold entries are the one that are statistically significant different

### The immune cell composition is altered in HCMV seropositive sepsis patients

The number of TEMRA (CD45RA^+^CCR7^−^CD27^−^CD28^−^) CD8^+^ T-cells was significantly higher in HCMV seropositive patients (79 cells per µL vs. 24 cells per µL HCMV seropositive vs seronegative, respectively, Man–Whitney U test: *p* < 0.001). In addition, we found the number of NKT-cells to be significantly higher in HCMV seropositive patients (48 cells per µL vs. 13 cells per µL, seropositive vs seronegative, respectively, Mann–Whitney *U* test: *p* < 0.001). Other cell population were not significantly different between the two groups when adjusted for multiple comparisons (Table 4).

**Table 4:** Immunophenotyping of sepsis patients by HCMV serostatus. *P*-values of *p* < 0.002 were considered significant as determined by Bonferroni correction

	HCMV seronegative	HCMV seropositive	p-value
***n***	54	91	
Leucocytes median [IQR]	14,159 [13508]	11,805 [9073]	0.245
Granulocytes median [IQR]	11,883 [12389]	9616 [8824]	0.273
Lymphocytes median [IQR]	738 [846]	646 [738]	0.167
CD3^−^ CD56^−^ cells median [IQR]	193 [260]	142 [178]	0.072
B-cells median [IQR]	106 [174]	63 [108]	0.065
NK cells median [IQR]	97 [87]	59 [79]	0.012
CD56^bright^ cells median [IQR]	4 [5]	3 [8]	0.984
CD56^dim^ cells median [IQR]	81 [81]	45 [82]	0.007
**NKT cells median [IQR]**	**7 **[11]	**22 **[45]	** < 0.001**
T-cells median [IQR]	499 [523]	365 [441]	0.275
CD4^+^ T-cells median [IQR]	364 [411]	218 [330]	0.032
CD4^+^ CM median [IQR]	101 [115]	59 [83]	0.034
CD4^+^ EM median [IQR]	78 [109]	59 [92]	0.237
CD4^+^ naïve median [IQR]	138 [208]	78 [161]	0.034
CD4^+^ TEMRA median [IQR]	8 [21]	8 [21]	0.894
CD4^−^ CD8^−^ T-cells median [IQR]	14 [21]	11 [16]	0.132
CD8^+^ T-cells median [IQR]	84 [123]	106 [165]	0.062
CD8^+^ CM median [IQR]	21 [35]	24 [44]	0.314
CD8^+^ EM median [IQR]	19 [26]	16 [35]	0.289
CD8^+^ naïve median [IQR]	27 [57]	39 [71]	0.095
**CD8**^**+**^** TEMRA median [IQR]**	**11 **[28]	**34 [89]**	** < 0.001**
CD14^+^ monocytes median [IQR]	1586 [2651]	1051 [2362]	0.072
classical monocytes median [IQR]	1375 [2328]	872 [2233]	0.067
intermediate monocytes median [IQR]	24 [48]	16 [43]	0.479
non-classical monocytes median [IQR]	8 [21]	4 [8]	0.031

Bold entries are the one that are statistically significant different

### The HCMV serostatus is associated with the immune reaction during sepsis and its impact on mortality

**Fig. 2:**
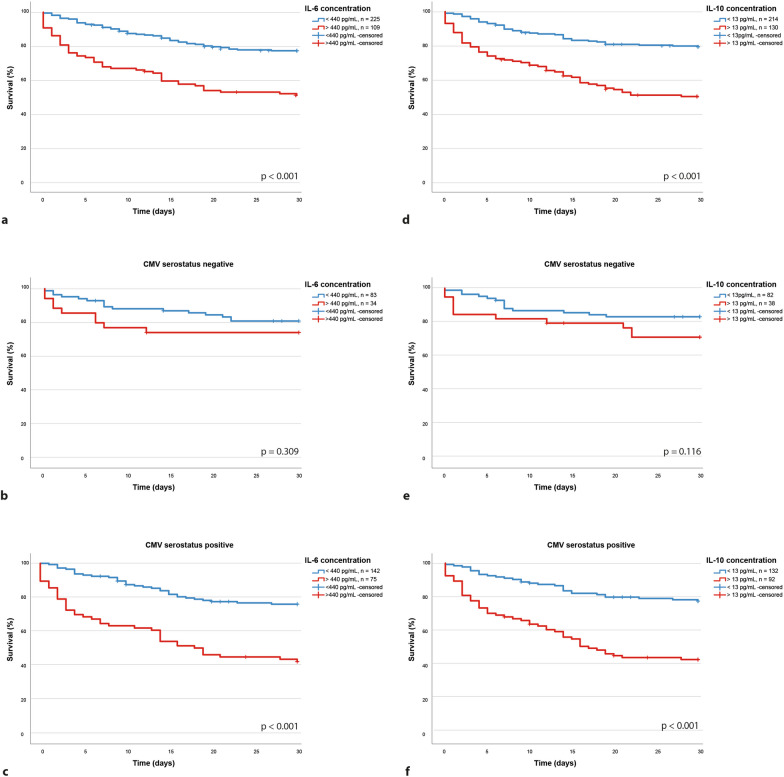
The prognostic value of IL-6 (**a**–**c**) as well as IL-10 (**d**–**f**) in our cohort was limited to the CMV seropositive group and was absent in the seronegative cohort

In our study cohort, high levels of IL-6 and IL-10 at the onset of sepsis (day of study inclusion) were significantly associated with the 30-day mortality (Fig. 2a, d) as could be expected based on the literature [7–9]. Surprisingly, this association was strongly enhanced in HCMV-seropositive (Fig. 2c, f) patients but was not significant in HCMV-seronegative patients (Fig. 2b, e).

**Fig. 3:**
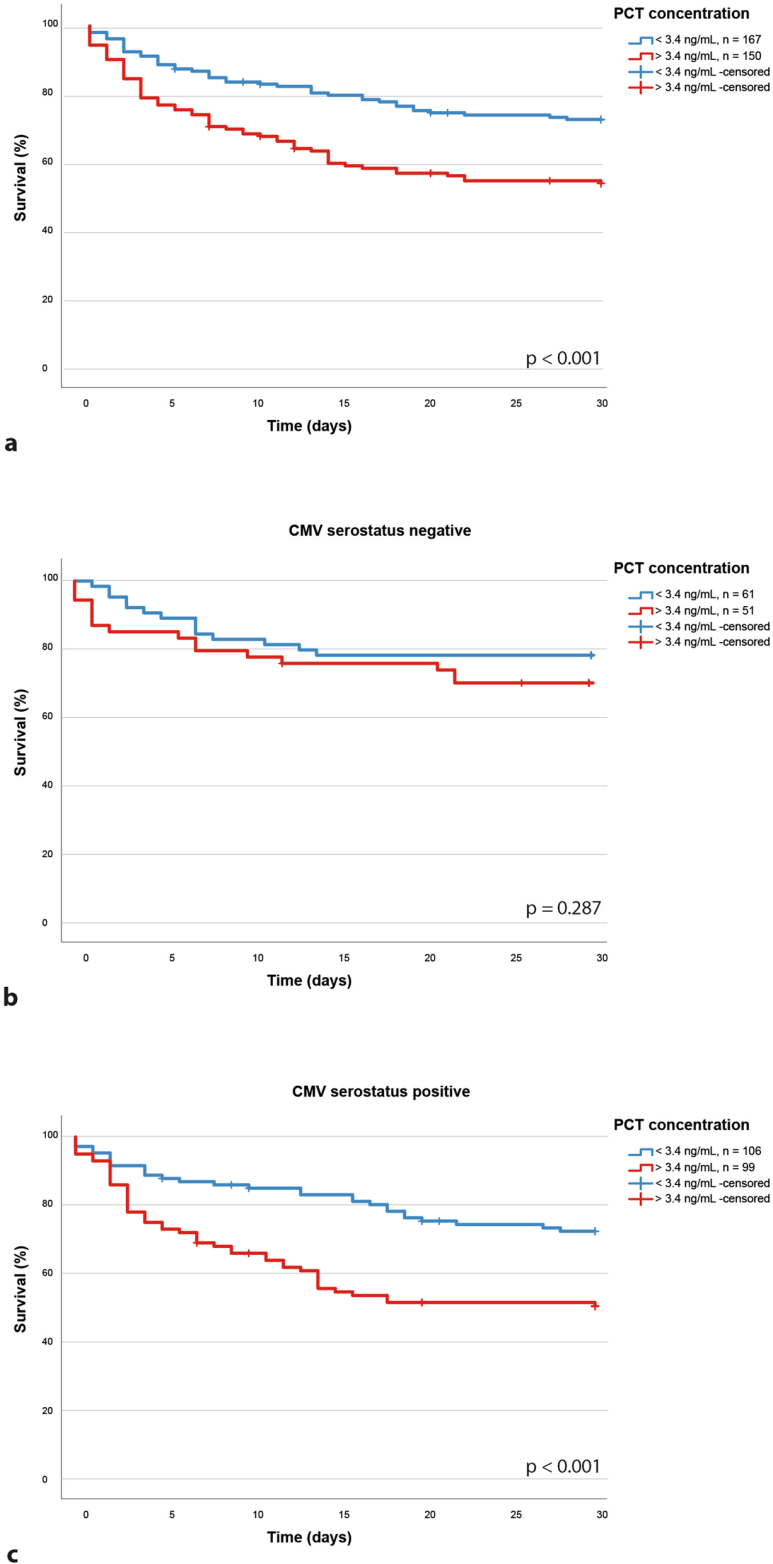
While procalcitonin, a clinically very relevant biomarker, had a predictive effect in the entire cohort (**a**), we could not observe a predictive value in the HCMV-seronegative patients (**b**). PCT is only of predictive value in the HCMV-seropositive cohort (**c**)

**Fig. 4:**
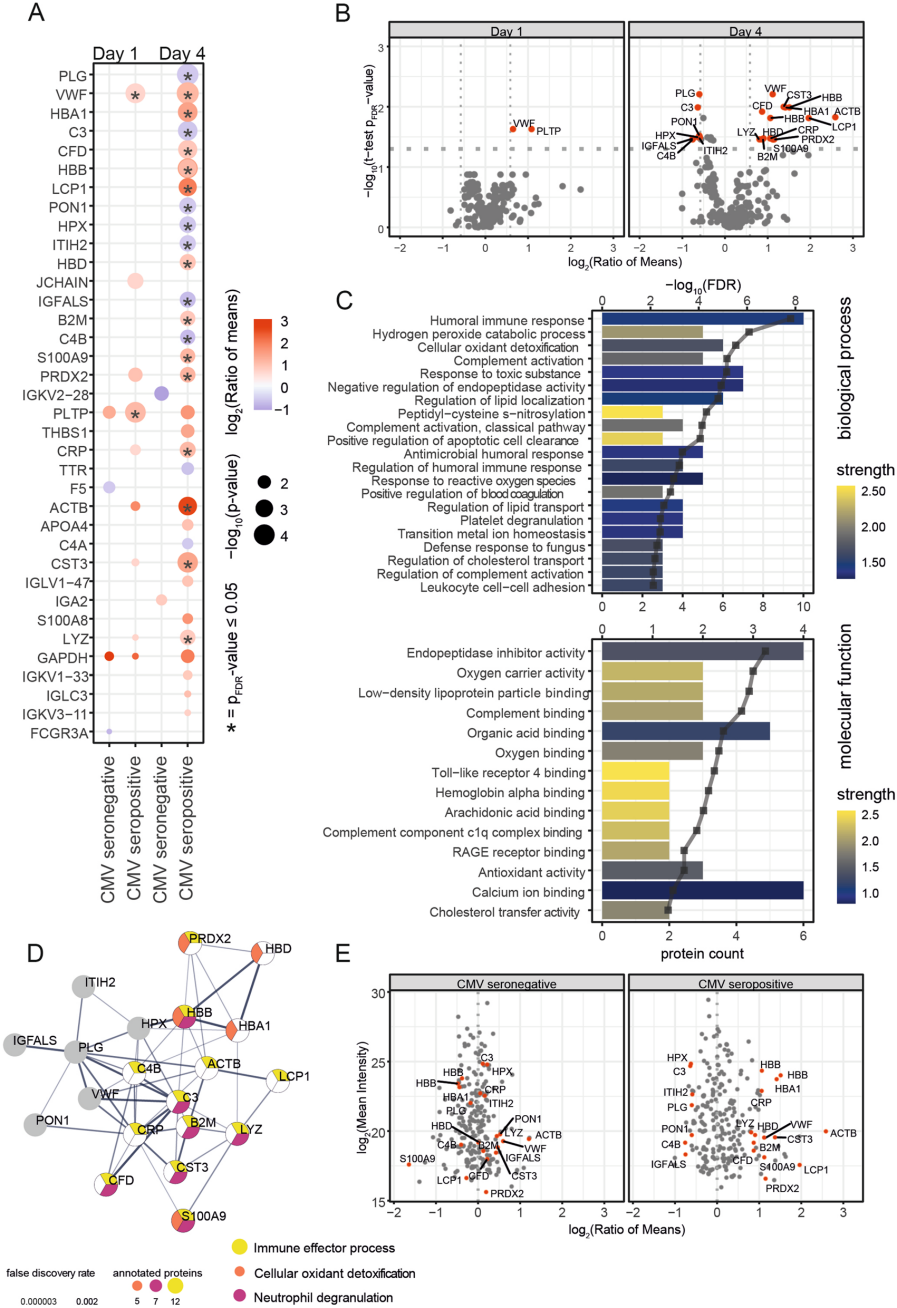
The plasma proteomics analysis according to survival. **a** Dot-plot illustrating the statistical analysis of *n* = 66 HCMV seronegative and *n* = 105 seropositive patients at days 1 and 4 (Student’s *t*-test; two-sided, unequal variances). **b** Volcano plot illustrating plasma proteome quantitation for seropositive patients and days 1 and 4 (*p*-values corrected according to Benjamini-Hochberg). Significantly regulated proteins (p_FDR_ value ≤ 0.05, absolute ratio of means ≥ 1.5) highlighted in red and labeled with gene names. **c** Gene ontology enrichment analysis of proteins regulated in seropositive patients at day 4 (*p*-value ≤ 0.05, absolute ratio of means ≥ 1.5). **d** Network representation of regulated proteins passing the p_FDR_ threshold of 0.05. Selected enriched ontologies (biological process) highlighted. **e** Scatter plots illustrating plasma proteome dynamics at day 4 for HCMV seronegative and seropositive patients. Proteins significantly regulated in seropositive patients are highlighted in both plots

Using the Youden’s Index we could define a cut-off of 440 pg/mL for IL-6 (52% sensitivity and 76% specificity) and 13.5 pg/mL for IL-10 (59% sensitivity and 72% specificity) as points of best discrimination in the entire cohort. In seropositive patients these cut-offs were associated with a more than three-fold higher 30-day mortality (69% vs. 24%; HR 3.250; 95% CI 2.075–5.090; *p* < 0.001) for IL-6 (Fig. 2c) and a similar increase in 30-day mortality for IL-10 (58% vs. 23%; HR 3.407; 95% CI 2.173–5.340; *p* < 0.001; Fig. 2f). In HCMV-seronegative patients, we could not observe a significant association with the 30-day mortality (*p* = 0.309 for IL-6; Fig. 2b and p = 0.116 for IL-10; Fig. 2e). Furthermore, this effect proved to be independent of age and admission SOFA score in seropositive patients (*p* = 0.005 for IL-6 and *p* = 0.002 for IL-10) while it could not be observed in the seronegative cohort (*p* = 0.476 for IL-6 and *p* = 0.913 for IL-10). Further analysis of the common routine marker PCT lead to a similar finding. The predictive value regarding the 30-day survival, illustrated in Kaplan–Meier plots, could be shown in the entire patient cohort (48% vs. 27%, *p* < 0.001; HR: 2.054, 95% CI 1.404–3.004, Fig. 3a) and even more in the HCMV-seropositive patients (56% vs. 29%, *p* < 0.001 HR 2.402, 95% CI 1.537–3.754, Fig. 3c) but was again not significant in the CMV-seronegative patients (32% vs. 24%, *p* = 0.339, HR: 1.429, 95% CI 0.680–3.004, Fig. 3b). When evaluating the plasma proteome dynamics, we found similar results. Overall, only few proteins were significantly regulated when all analyzed samples were taken into account (Additional file 1: Figure S1, Additional file 2: Figure S2). However, the separate analysis of seropositive patients with regard to mortality revealed a range of proteins differentially regulated between the deceased and surviving patients (Fig. 4a, b). These were mainly related to immune effector processes, both on a cellular and humoral level (Fig. 4c). Key proteins, such as C-reactive protein (CRP), lysozyme (LYZ), or von Willebrand factor (vfW), were found to be regulated in patients with an adverse prognosis. The Immunomodulatory S100A9 protein which is released from neutrophils during degranulation and other danger signals like Peroxiredoxin 2 (PRDX2) and actin (ACTB) were also elevated (Fig. 4d). In contrast, no significantly regulated proteins were found in HCMV-seronegative patients (Fig. 4e, Additional file 2: Figure S2). This further strengthens the hypothesis that the host immune response plays a different role in HCMV-seropositive patients than in seronegative patients.

## Discussion

With this study we provide a novel and alternative view on the ongoing debate, whether or not HCMV re-activation is detrimental for the outcome of sepsis. We propose that HCMV latency, rather than re-activation could be the real culprit. HCMV latency refers to the period when the virus is present in a silent, inactive form within the host. During latency, infectious virus particles are not produced by the virus. Instead, the viral genome is maintained within the host cell's nucleus, and only a limited set of viral genes is expressed at low levels [32]. This limited gene expression helps the virus evade the immune system and persist in the host. Because of absence of virions in the latent state the best way to detect HCMV latency is a positive HCMV IgG serostatus. Our data demonstrate a strong association between this HCMV serostatus and sepsis survival. HCMV seropositivity was highly predictive for worsened outcomes in sepsis. This suggests that HCMV serostatus should be considered when evaluating the predictive value of common biomarkers and cytokines in sepsis. As latent HCMV infections, which are the reason for HCMV seropositivity, affect the majority of mankind [14], our findings are relevant to most adult sepsis patients. In this context, the impact of HCMV latency on the complex interplay of human immunity based on non-replicating viruses has been discussed [18, 33, 34]. Brodin et al. even go so far as to call HCMV latency the strongest non-heritable immune modulator in humans [35]. In vitro inflammation in HCMV-infected human macrophages resulted in an increased expression of toll-like receptors TLR-4 and TLR-5 at the cell surface, potentiating the susceptibility and downstream activation of the cellular response to LPS stimulation [28]. In vivo, after vaccination against influenza, the HCMV-seropositive individuals presented an enhanced antibody response and elevated circulating IFN-γ levels compared with the HCMV-seronegative individuals [36]. In contrast to this HCMV seropositive patients exhibit a lower number of Influenca specific CD8 T-cells than seronegative patients [37]. Overall, these findings showed a modified inflammatory response in HCMV-seropositive individuals. Usually these changes are attributed to re-activation of HCMV, a dogma that exists in intensive care medicine for some time. In line with this, while HCMV reactivation has been intensely studied in sepsis patients for more than 30 years [13, 38, 39], only few studies investigated the HCMV serostatus and its relevance in infectious diseases. One retrospective study [40] that focused on critically ill patients in general could not demonstrate a relevant impact on the patients’ survival. However, as that patient cohort only consisted of around 60% sepsis patients (SEPSIS-1 definition), only limited conclusions could be drawn regarding sepsis. To our knowledge, the impact of the HCMV serostatus on patients suffering from sepsis (according to SEPSIS-3) has not yet been investigated.

Therefore, we performed a post hoc analysis of the multicentric SepsisDataNet.NRW study that prospectively enrolled patients suffering from sepsis. In this study cohort, we found the expected proportion of HCMV-positive patients (63%) and could for the first time show that these patients had a worse prognosis than the HCMV seronegative patients. Of note, seropositive patients were slightly older and sicker than seronegative patients, which could theoretically explain the observed effects on survival as confounders. From a different perspective, one could speculate that the higher severity of the disease might also be a consequence of HCMV latency.

As reactivation might also be an important confounder, we analyzed the HCMV reactivation both by qPCR and by measuring IgM and could not detect a relevant number of reactivating patients even until Day 8. Due to the post hoc nature of our study, we cannot make a definite statement on the impact of later HCMV reactivation. However, a recent study evaluating HCMV reactivation in sepsis patients determined the median time to reactivation at seven days with a total reactivation of 18.3% in their cohort [41]. On the other hand, other studies found a meaningful reactivation to occur by Day 21 [20]. This high heterogeneity makes it difficult to accurately rule out HCMV reactivation as a confounder. However, as the effect of HCMV serostatus on patient survival is already significant at Day 8 (*p* = 0.014), we postulated nonetheless that CMV reactivation, even if it is expected later in the course of the disease, would not change our findings. Thus, we concluded that HCMV reactivation is not an important confounder in our cohort in relation to survival. Based on our findings, we conclude that the impact of HCMV latency on the immune reaction is at least partially responsible for the observed effects of the serostatus on survival. Obviously, there are a range of possible confounders which, due to the post-hoc nature of this analysis we could not correct for. In the SepsisDataNet.NRW study the socio-economic status, which is a major contributor to HCMV seropositivity was not assessed, neither were other possible confounders such as ethnicity. However, to our knowledge these confounders do not influence survival in sepsis, so they are unlikely to explain our findings. Another possible confounder for HCMV serostatus as well as an altered immune response is age. However, in our Cox Regression analysis the effect of the cytokines was independent of age and disease severity (SOFA score). Additionally, it is important to acknowledge that the representation of HCMV seronegative patients in any representative cohort, including ours, is naturally limited due to the statistical distribution. Consequently, the power of the HCMV seronegative cohort is lower. However, the effect size observed in the seropositive cohort is substantial enough to be detectable even in the seronegative cohort. Therefore, we are confident that the impact of the immune reaction is largely limited to the seropositive cohort.

In order to investigate the implications of HCMV-induced changes to the human immunity, we compared the cytokine levels of seropositive- and -negative patients during the study inclusion period. Only the IL-8 serum concentration was found to be significantly different at Day 1. This is consistent with previous literature suggesting the elevated IL-8 levels in HCMV-seropositive patients [28]. Interestingly, as seropositive patients show a higher number of NKT-cells, one could expect an increase of IFN-γ [42]. However, as cytokines are produced by many different cells it is not un-surprising to not see correlations with the HCMV serostatus. While the serum levels of all other measured cytokines as well as a mass spectrometry-based analysis of the plasma proteome showed no significant difference with regard to the HCMV serostatus, we indicated that the expected predictive value of IL-6 [7] and IL-10 [43] on the patients’ survival was associated with the HCMV serostatus. Serum PCT showed an analogous association. For all tested cytokine-concentrations, the best discriminating cutoff-value was determined calculating the Youden-Index. Beyond this, our cutoff values for IL-6 and IL-10 resemble reported values [44, 45].

This is surprising considering that the predictive value for IL-6, IL-10, and other immunological marker proteins is described in many studies in prestigious journals [7–9]. Our data do not contradict these earlier findings but rather suggest that the predictive power of those cytokines is based solely on HCMV-positive individuals, which frequently accounts for the major parts of the study population. This also appears notable as the interpretation of these often-used markers might have to be seen in a different perspective from now on. In line with this, we found multiple differentially abundant plasma proteins, such as CRP, vWF [46], or lysozyme [47], in HCMV-seropositive patients to be associated with the survival status, which is not observed in HCMV-seronegative individuals. These data show that protein networks related to reactive oxygen species, complement activation and apoptotic cell clearance are stronger activated in HCMV seropositive patients succumbing to the disease. One possible implication from these data is that immunomodulation (e.g., using hemadsorption [48] or anti IL-6 antibodies [49]) might only be proven beneficial for HCMV-seropositive patients.

From a clinical point of view, we also considered the common biomarker serum procalcitonin. Interestingly, we found a similar effect to the one seen in cytokines, restricting the predictive power of PCT [50] to only CMV-seropositive patients. In light of this, we propose to re-evaluate common clinical biomarkers in sepsis with regard to HCMV serostatus. Although our data are retrospective in nature, it is tempting to speculate on possible mechanisms, including a HCMV latency-related change in the immune reaction. However, further research will be needed to determine the underlying relationships.

## Conclusion

In a large multicentric cohort of prospectively recruited sepsis patients with an expected rate of HCMV-seropositive individuals, we observed a notable association between HCMV latency, as indicated by HCMV IgG seropositivity, and sepsis mortality. This appears to be primarily attributed to the differing impact of immunological molecules, such as cytokines, on the survival of seropositive and seronegative patients. While the intensity of the inflammatory response remains a significant factor in sepsis prognosis, it is noteworthy that this impact is closely linked to the HCMV serostatus. Additional research is warranted and may prompt a re-evaluation of the prognostic value of established biomarkers like procalcitonin in relation to HCMV serostatus. In a clinical context, it is worth considering the early assessment of patients' HCMV serostatus, which could inform clinical decision-making regarding adjunctive therapies such as cytokine modulation or therapeutic immunoglobulins.

### Supplementary Information

**Additional file 1**.** Fig. S1**: Volcano plot illustrating the statistical analysis of plasma proteomics data for days 1 and 4. CMV serostatus positive and negative patients were tested for significant differences using student’s t-test (two-sided, unequal variances). False discovery rate-adjusted (according to Benjamini-Hochberg)* p*-values plotted against ratios of mean intensities (positive/negative). For day 1, no significant changes were observed. For day 4,one protein, highlighted in red and labeled with its gene name, passed the significance threshold of a pFDR-value ≤ 0.05 and an absolute ratio of means ≥ 1.5.

**Additional file 2**. ** Fig. S2**: Statistical analysis of plasma proteomics data separately for days 1 and 4 as well as all patients, CMV serostatus positive and negative patients, respectively. Differences between deceased and survived patients were evaluated by student’s * t*-test (two-sided, unequal variances). Volcano plots illustrating pFDR-values (adjusted according to Benjamini-Hochberg) plotted against ratios of mean protein intensities (Exitus/Survival). Proteins passing the significance threshold of a pFDR-value ≤ 0.05 and an absolute ratio of means ≥ 1.5 highlighted in red and labeled with gene names.

**Additional file 3. Table S1**:  Statistical analysis of quantitative plasma proteomics data - CMV serostatus-specific analysis.

**Additional file 4. Table S2**: Statistical analysis of quantitative plasma proteomics data - survival-specific analysis.

**Additional file 5. Table S3**: a) Longitudinal cytokine concentration in serum of HCMV seronegative sepsis patients. b) Longitudinal cytokine concentration in serum of HCMV seropositive patients.

## Data Availability

Data on which the conclusions are drawn are available upon reasonable request from the corresponding author.
