# Supplementary figures and images for: Human cytomegalovirus seropositivity is associated with reduced patient survival during sepsis
Source: Crit Care. 2023 Oct 31;27:417. doi: 10.1186/s13054-023-04713-1 (PMC10619294; doi:10.1186/s13054-023-04713-1)

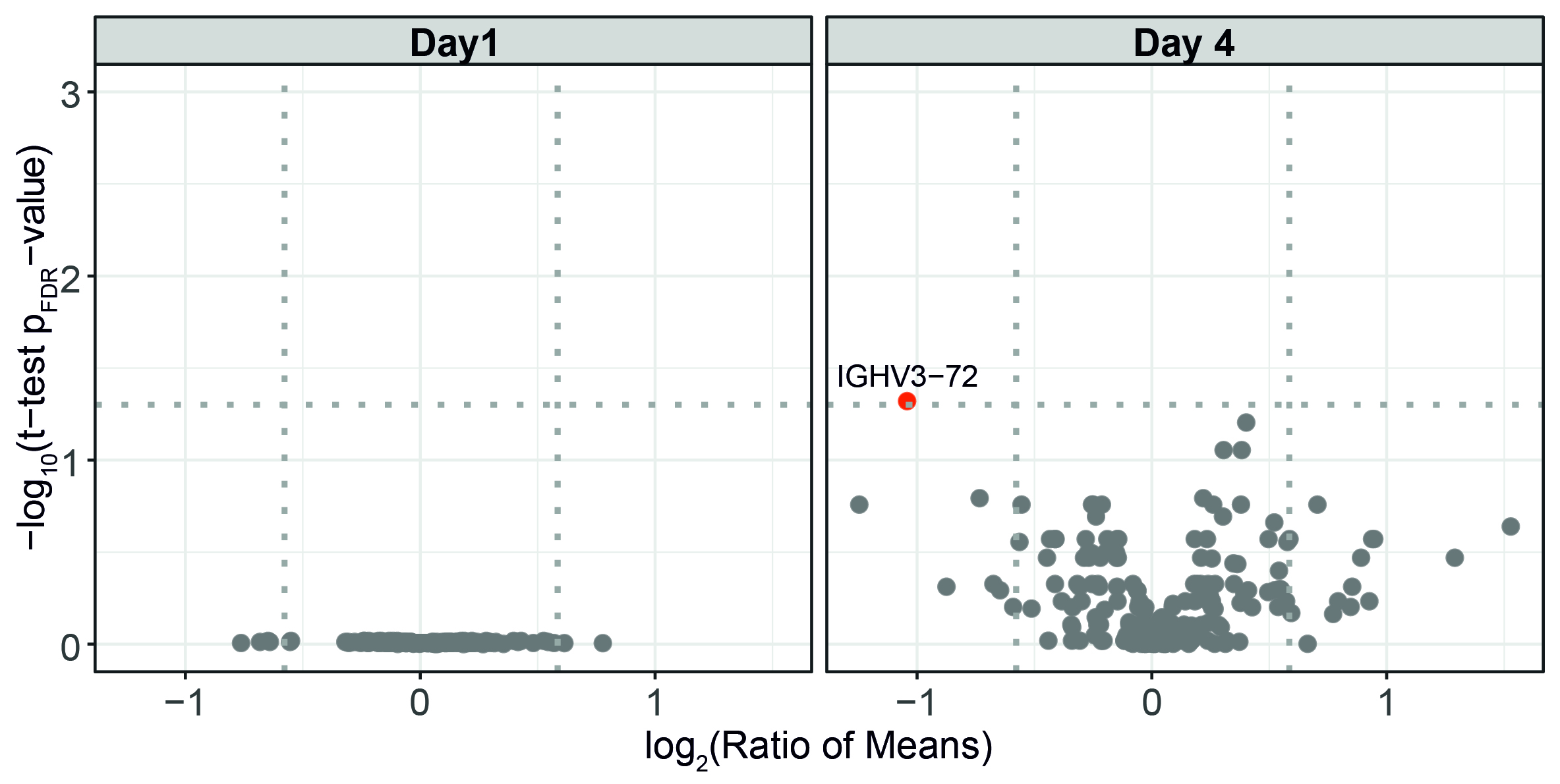

Supplement: Supplementary file 1 — Additional file 1. Fig. S1: Volcano plot illustrating the statistical analysis of plasma proteomics data for days 1 and 4. CMV serostatus positive and negative patients were tested for significant differences using student’s t-test (two-sided, unequal variances). False discovery rate-adjusted (according to Benjamini-Hochberg) p-values plotted against ratios of mean intensities (positive/negative). For day 1, no significant changes were observed. For day 4,one protein, highlighted in red and labeled with its gene name, passed the significance threshold of a pFDR-value ≤ 0.05 and an absolute ratio of means ≥ 1.5. [file 13054_2023_4713_MOESM1_ESM.jpg]

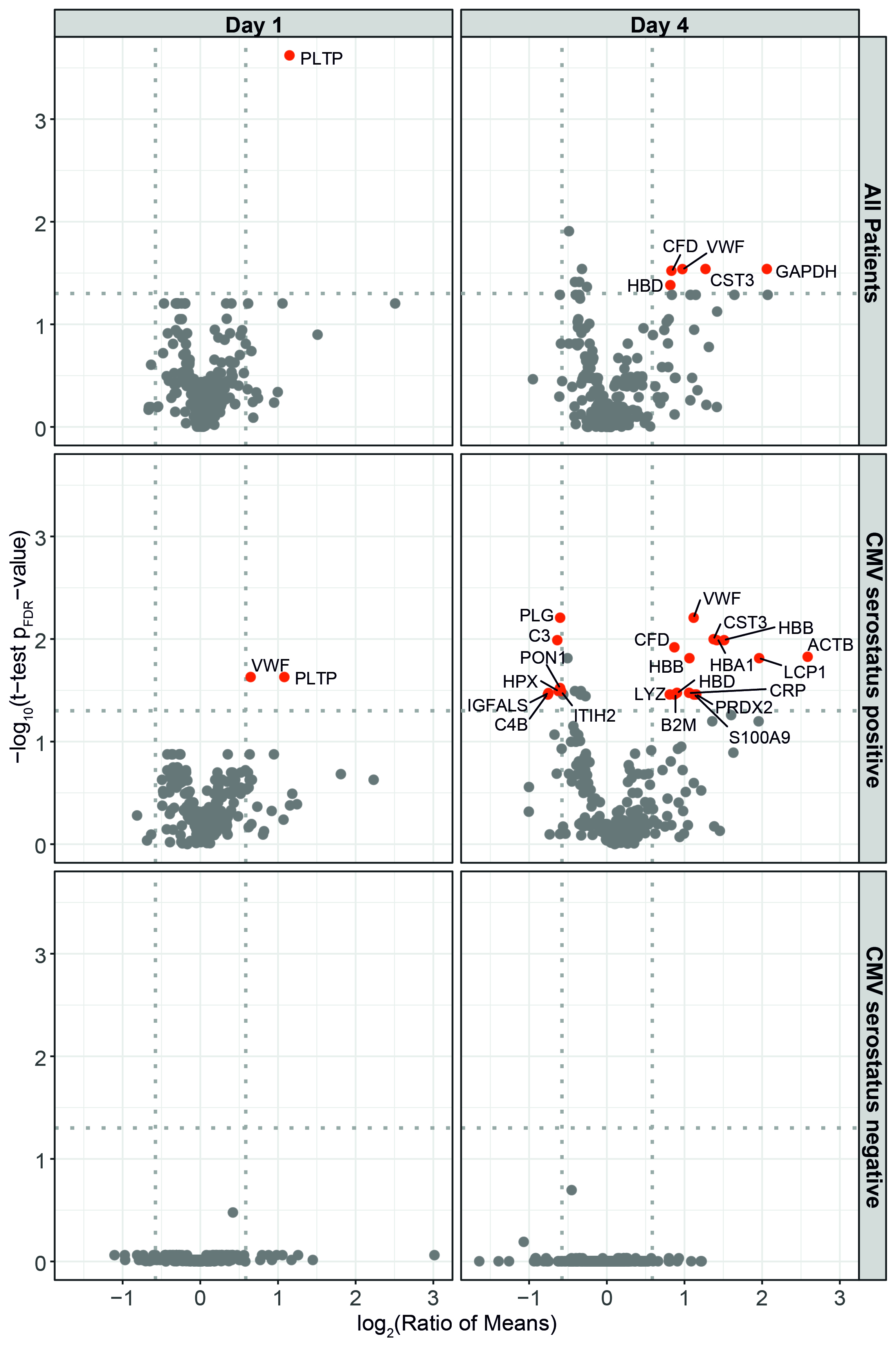

Supplement: Supplementary file 2 — Additional file 2. Fig. S2: Statistical analysis of plasma proteomics data separately for days 1 and 4 as well as all patients, CMV serostatus positive and negative patients, respectively. Differences between deceased and survived patients were evaluated by student’s t-test (two-sided, unequal variances). Volcano plots illustrating pFDR-values (adjusted according to Benjamini-Hochberg) plotted against ratios of mean protein intensities (Exitus/Survival). Proteins passing the significance threshold of a pFDR-value ≤ 0.05 and an absolute ratio of means ≥ 1.5 highlighted in red and labeled with gene names. [file 13054_2023_4713_MOESM2_ESM.jpg]
