# Supplementary material for: Human cytomegalovirus seropositivity is associated with reduced patient survival during sepsis
Source: Crit Care. 2023 Oct 31;27:417. doi: 10.1186/s13054-023-04713-1 (PMC10619294; doi:10.1186/s13054-023-04713-1)
Supplement: Supplementary file 5 — Additional file 5. Table S3: a) Longitudinal cytokine concentration in serum of HCMV seronegative sepsis patients. b) Longitudinal cytokine concentration in serum of HCMV seropositive patients. [file 13054_2023_4713_MOESM5_ESM.docx]

Supplementary Table 4a: Longitudinal cytokine concentration in serum of HCMV seronegative sepsis patients.

| **CMV seronegative** | **Day 1** | **Day 4** | **Day 8** |
| --- | --- | --- | --- |
| **n** | 117 | 96 | 63 |
| **IL-1B median [IQR]** | 5.1 [1.1-9.0] | 5.1 [2.9-9.5] | 4.9 [2.8-11.5] |
| **IFN-α median [IQR]** | 1.6 [1.1-2.4] | 1.4 [0.0-2.2] | 1.7 [1.1-2.1] |
| **IFN-γ median [IQR]** | 4.9 [0.3-10.2] | 5.2 [1.2-11.3] | 5.8 [2.4-10.6] |
| **TNF-α median [IQR]** | 5.3 [0.0-9.3] | 6.4 [0.0-9.3] | 6.3 [0.0-12.0] |
| **MCP-1 median [IQR]** | 248.5 [143.9-466.7] | 216.1 [141.2-320.7] | 172.7 [116.4-292.7] |
| **IL-6 median [IQR]** | 170.1 [71.1-481.2] | 60.0 [22.3-134.4] | 52.6 [17.7-117.6] |
| **IL-8 median [IQR]** | 61.0 [26.9-121.8] | 34.4 [18.6-81.9] | 61.0 [31.6-98.0] |
| **IL-10 median [IQR]** | 6.7 [2.2-17.7] | 3.0 [0.0-7.6] | 7.9 [2.3-11.5] |
| **IL-12 median [IQR]** | 2.9 [0.0-4.4] | 2.7 [1.8-4.0] | 2.3 [0.0-4.7] |
| **IL17 median [IQR]** | 0.5 [0.3-0.8] | 0.5 [0.3-0.8] | 0.6 [0.4-0.9] |
| **IL-18 median [IQR]** | 225.8 [115.7-548.2] | 234.9 [132.8-462.0] | 212.7 [87.9-460.7] |
| **IL-23 median [IQR]** | 10.7 [0.0-30.3] | 6.6 [0.0-19.8] | 10.0 [0.0-30.1] |
| **IL-33 median [IQR]** | 25.6 [12.0-47.5] | 21.9 [0.0-41.9] | 26.7 [13.0-46.5] |

Supplementary Table 4b: Longitudinal cytokine concentration in serum of HCMV seropositive patients.

| **CMV seropositive** | **Day 1** | **Day 4** | **Day 8** |
| --- | --- | --- | --- |
| **n** | 217 | 138 | 88 |
| **IL-1B median [IQR]** | 5.1 [2.7-8.9] | 5.1 [7.3] | 5.1 [8.9] |
| **IFN-α median [IQR]** | 1.6 [1.2-2.9] | 1.6 [1.1] | 1.5 [0.8] |
| **IFN-γ median [IQR]** | 4.0 [0.0-10.2] | 3.7 [8.6] | 3.7 [8.7] |
| **TNF-α median [IQR]** | 6.3 [0.0-11.3] | 5.9 [11.0] | 6.3 [12.0] |
| **MCP-1 median [IQR]** | 306.0 [150.4-570.5] | 235.3 [241.1] | 234.9 [157.4] |
| **IL-6 median [IQR]** | 259.9 [64.4-654.7] | 59.2 [134.9] | 63.3 [115.2] |
| **IL-8 median [IQR]** | 84.2 [37.0-199.6] | 51.1 [101.0] | 67.2 [100.7] |
| **IL-10 median [IQR]** | 8.2 [2.3-25.6] | 7.5 [17.3] | 6.6 [11.2] |
| **IL-12 median [IQR]** | 2.8 [1.5-4.4] | 2.6 [3.7] | 2.4 [4.0] |
| **IL17 median [IQR]** | 0.5 [0.3-0.9] | 0.6 [0.6] | 0.5 [0.5] |
| **IL-18 median [IQR]** | 345.1 [153.3-771.4] | 446.3 [827.5] | 410.0 [743.0] |
| **IL-23 median [IQR]** | 6.1 [0.0-25.4] | 8.0 [26.1] | 8.7 [30.2] |
| **IL-33 median [IQR]** | 22.5 [9.7-46.5] | 24.0 [36.9] | 25.6 [43.9] |
